# Uncovering the hidden marine sponge microbiome by applying a multi-primer approach

**DOI:** 10.1038/s41598-019-42694-w

**Published:** 2019-04-17

**Authors:** Qi Yang, Christopher M. M. Franco, Wei Zhang

**Affiliations:** 1 Centre for Marine Bioproducts Development, College of Medicine and Public Health, Flinders University, Adelaide, South Australia 5042 Australia; 2 Center for Marine Drugs, State Key Laboratory of Oncogene and Related Genes, Department of Pharmacy, Renji Hospital, School of Medicine, Shanghai Jiao Tong University, Shanghai, 200127 China

**Keywords:** Environmental microbiology, Microbial ecology

## Abstract

Marine sponges (phylum Porifera) are hosts to microorganisms that make up to 40–60% of the mesohyl volume. The challenge is to characterise this microbial diversity more comprehensively. To accomplish this, a new method was for the first time proposed to obtain sequence coverage of all the variable regions of the 16S rRNA gene to analyze the amplicon-based microbiomes of four representative sponge species belonging to different orders. The five primer sets targeting nine variable regions of the 16S rRNA gene revealed a significant increase in microbiome coverage of 29.5% of phylum level OTUs and 35.5% class level OTUs compared to the community revealed by the commonly used V4 region-specific primer set alone. Among the resulting OTUs, 52.6% and 61.3% were unaffiliated, including candidate OTUs, at the phylum and class levels, respectively, which demonstrated a substantially superior performance in uncovering taxonomic ‘blind spots’. Overall, a more complete sponge microbiome profile was achieved by this multi-primer approach, given the significant improvement of microbial taxonomic coverage and the enhanced capacity to uncover novel microbial taxa. This multi-primer approach represents a fundamental and practical change from the conventional single primer set amplicon-based microbiome approach, and can be broadly applicable to other microbiome studies.

## Introduction

Marine sponges (phylum Porifera), sessile filter feeders, form symbiotic relationships with complex communities of microorganisms^1^. Up to 40–60% of the tissue volume of certain sponge species consists of microorganisms with a density exceeding 10^9^ microbial cells per ml of sponge tissue; orders of magnitude greater than that found in surrounding seawater or sediment^2–5^.

PCR-based amplification and sequencing of the 16S rRNA gene contributes greatly to our understanding of the microbial world^6^, including complex sponge microbiomes^7^. This gene has nine hypervariable regions (V1–V9) that are flanked by conserved stretches^8^. In principle, all the nine hypervariable 16S rRNA gene regions could be targeted for microbial profiling and indeed different variable regions have been investigated using a single primer set^4,9–13^. This single primer set approach employing region-specific primers is reflected in all the large-scale microbiome studies, including the Earth Microbiome Project (EMP) using V4^14^; the Human Microbiome Project (HMP) using V3V5^15^, and the Sponge Microbiome Project, as part of EMP, using V4^7^. The choice of specific region was often due to its superior performance than the others, even with the well-documented knowledge of significant primer bias on both community richness and evenness when targeting different regions^9,10,16–18^. A number of studies have searched for the optimum variable region(s) that can be amplified by a single primer pair^19–22^. This is often justified on cost, ignoring the experimental evidence of high dependence of microbial diversity metrics on region choice from these comparative studies (e.g.^9,18^). This single primer approach excludes a substantial component of the microbiome without an understanding of the quantifiable impact that leads to a diminished and biased finding, even though some researchers recommend that other regions need to be surveyed^16^.

The purposes of this study were to test the hypotheses that the primer sets targeting different variable regions of the 16S rRNA gene indeed reveal vastly different parts of the microbiome; and that the combination of multi-primers covering all the regions would uncover a more complete profile of the sponge microbiome. An improved multi-primer approach, with five primer sets specific to regions of V1V3, V4, V4V5, V5V8, and V6V9 covering the full length of the 16S rRNA gene, was applied in an amplicon-based metagenomic sequencing on Illumina MiSeq. In order to test the hypotheses the datasets generated from all the five primer sets from each sponge species were analyzed to reveal the microbial communities of four representative sponge species belonging to four different orders.

## Results

### Methodology validation

The reliability of the technical replicates among the multiple sequencings was validated by the consistency of the microbial community between the theoretical microbial composition and the measured composition in each sequencing run of the quality control ZymoBIOMICS™ Microbial Community Standard (Cellular Standard and DNA Standard) (Supplementary Fig. S1). All the five primer sets were able to detect the known microbiota within the mock community. The microbiota recovery efficiency varied with the region-specific primer sets (Supplementary Fig. S2). The primer set for regions V5V8 had a better recovery efficiency than V4 and V4V5 primer sets that showed comparable capacity, followed by V1V3 and V6V9. The weighted UniFrac metric of beta-diversity analysis showing on Principal Coordinates Analysis (PCoA) plots demonstrated highly matched technical replicates between PCR amplifications from the same sponge individual revealed by each of the five primer sets for 16S rRNA gene regions V1-V9 (Supplementary Fig. S3a-e). The divergences between the three biological replicates belonging to the same sponge species was evident (Supplementary Fig. S3a–e), however, these differences were reduced significantly when the five datasets generated from five primer sets were analyzed together (Supplementary Fig. S3f). The sequencing reads generated by five primer sets for respective 16S rRNA gene regions (V1V3, V4, V4V5, V5V8, and V6V9) further confirmed the sequencing reads consistency between the technical replicates and between biological replicates (Supplementary Table S1). The consistency of both the sequencing reads and the beta-diversity validated the use of the highest sequence read among the replicates for each primer set for each sponge species was the best representative selection to be employed for the purpose of this study with minimum bias.

### Significant increase in revealing affiliated OTUs

**Figure 1:**
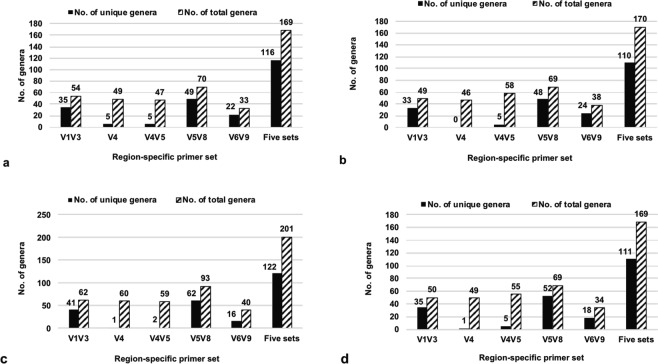
Number of unique and total affiliated (known & named) genera revealed by each region-specific primer set on Illumina MiSeq platform. The specific primers are for regions V1V3, V4, V4V5, V5V8, and V6V9 of 16S rRNA gene. The number of unique genera and the total number of genera using all five primer sets were shown as the last group in each chart. (**a**) Sponge *Aplysina archeri*; (**b**) Sponge *Halichondria okadai*; (**c**) Sponge *Igernella notabilis*; (**d**) Sponge *Tedania tubulifera* (see Supplementary Dataset S1).

Overall, 27 phylum-level affiliated microbial operational taxonomic units (OTUs) were revealed −9 more than using region V4 only (Table 1). In addition, 27 more class-level affiliated OTUs were obtained compared to the region V4 dataset (Table 2). Moreover, a comparison of the revealed OTUs at the genus level for the four different sponge species is shown in Fig. 1, in terms of the numbers of unique (found only with one primer set for a single sample) and total affiliated genera (known and named genera). The primers specific to regions V1V3, V4, and V4V5 revealed similar numbers of total genera (46–62 genera) from each of the four sponge species, the primers targeting the V5V8 region revealed the highest numbers of total genera (69–93 genera), and the V6V9 region revealed the lowest numbers of total genera, with 33–40 genera. However, the V1V3 region inferred 33–41 unique genera in comparison to 0–5 unique genera for each of the V4 and V4V5 regions. More profoundly, the V5V8 region revealed the highest numbers of unique genera (48–62 genera) that make up 42–51% of total unique genera generated from the combined dataset. Moreover, the V5V8 dataset also contributed the most (41–46%) to the total genera when the data from five primer sets were combined.

**Table 1:** The number of unaffiliated and affiliated OTUs at the phylum level derived from four sponge species revealed by five primer sets.

Primer set	*Aplysina archeri*			*Halichondria okadai*			*Igernella notabilis*			*Tedania tubulifera*			Total	
	Unaffi-liated^a^	Affili-ated	Unaffiliated/total^b^ (%)	Unaffiliated	Affiliated	Unaffiliated/total (%)	Unaffiliated	Affi-liated	Unaffiliated/total (%)	Unaffi-liated	Affi-liated	Unaffiliated/total (%)	Unaffiliated	Affiliated
Phylum level OTUs														
V1V3	4	15	4/(16 + 17)= 12.1%	9	20	9/(26 + 25)= 17.6%	9	19	9/(18 + 22)= 22.5%	6	14	6/(13 + 22)= 17.1%	11 (4)^c^	21 (12)^c^
V4	6	14	6/33 = 18.2%	24	15	24/(26 + 25)= 47.1%	12	14	12/(18 + 22)= 30.0%	11	12	11/(13 + 22)= 31.4%	26 (4)^c^	18 (11)^c^
V4V5	6	15	6/33 = 18.2%	11	18	11/(26 + 25)= 21.6%	8	18	8/(18 + 22)= 20.0%	8	16	8/(13 + 22)= 22.9%	14 (4)^c^	22 (13)^c^
V5V8	12	16	12/33 = 36.4%	10	24	10/(26 + 25)= 19.6%	10	19	10/(18 + 22)= 25.0%	10	18	10/(13 + 22)= 28.6%	21 (5)^c^	24 (15)^c^
V6V9	5	13	5/33 = 15.2%	5	13	5/(26 + 25)= 9.8%	4	14	4/(18 + 22)= 10.0%	2	14	2/(13 + 22)= 5.7%	5 (2)^c^	17 (9)^c^
Total	16 (0)^d^	17 (11)^d^	16/33 = 48.5%	26 (0)^d^	25 (10)^d^	26/(26 + 25)= 51.0%	18 (0)^d^	22 (10)^d^	18/(18 + 22)= 45.0%	13 (1)^d^	22(9)^d^	13/(13 + 22)= 37.1%	30 (8)^c^27 (17)^c^	30(2)^d^27 (12)^d^
Shared OTUs (%)	11/33 = 33.3%			10/51 = 19.6%			10/40 = 25.0%			10/35 = 28.6%			27/57 = 47.4%	

Total No. of unaffiliated phylum level OTUs/total No. of phylum level OTUs = 30/(30 + 27) = 52.6%.

^a^The unaffiliated OTUs include the candidate phyla and the unassigned OTUs; ^b^The total refers to the total No. of the unaffiliated OTUs and the affiliated OTUs revealed by five primer sets for one sponge species; ^c^The number in brackets refers to the No. of the shared phylum level OTUs among four sponge species; ^d^The number in brackets refers to the No. of the shared phylum level OTUs revealed by five primer sets.

**Table 2:** The number of unaffiliated and affiliated OTUs at the class level derived from four sponge species revealed by five primer sets.

Primer Set	*Aplysina archeri*			*Halichondria okadai*			*Igernella notabilis*			*Tedania tubulifera*		Total		
	Unaffi-liated^a^	Affili-ated	Unaffiliated/total^b^ (%)	Unaffi-liated	Affili-ated	Unaffiliated/total (%)	Unaffiliated	Affili-ated	Unaffiliated/total (%)	Unaffi-liated	Affili-ated	Unaffiliated/total (%)	Unaffiliated	Affiliated
Class level OTUs														
V1V3	17	24	17/(51 + 38)= 19.1%	27	39	27/(81 + 56)= 19.7%	19	33	19/(52 + 45)= 19.6%	20	28	20/(42 + 46)= 22.7%	38 (10)^c^	41 (18)^c^
V4	24	20	24/(51 + 38)= 27.0%	76	32	76/(81 + 56)= 55.5%	36	28	36/(52 + 45)= 37.1%	36	23	36/(42 + 46)= 40.9%	86 (15)^c^	38 (13)^c^
V4V5	25	24	25/(51 + 38)= 28.1%	36	34	36/(81 + 56)= 26.3%	28	33	28/(52 + 45)= 28.9%	22	27	22/(42 + 46)= 25.0%	53 (10)^c^	42 (18)^c^
V5V8	35	26	35/(51 + 38)= 39.3%	31	51	31/(81 + 56)= 22.6%	23	34	23/(52 + 45)= 23.7%	24	34	24/(42 + 46)= 27.3%	57 (12)^c^	55 (21)^c^
V6V9	13	25	13/(51 + 38)= 14.6%	13	27	13/(81 + 56)= 9.5%	15	28	15/(52 + 45)= 15.5%	4	23	4/(42 + 46)= 4.5%	18 (2)^c^	35 (14)^c^
Total	51 (2)^d^	38 (9)^d^	51/(51 + 38)= 57.3%	81 (4)^d^	56 (18)^d^	81/(81 + 56)= 59.1%	52 (2)^d^	45 (18)^d^	52/(52 + 45)= 53.6%	42 (1)^d^	46 (9)^d^	42/(42 + 46)= 47.7%	103 (23)^c^ 103 (6)^d^	65 (31)^c^ 65 (22)^d^
Shared OTUs (%)	11/89 = 12.4%			22/137 = 16.1%			20/97 = 20.6%			10/88 = 11.4%			65/168 = 38.7%	

Total No. of unaffiliated class level OTUs/total No. of class level OTUs = 103/(103 + 65) = 61.3%.

^a^The unaffiliated OTUs include the candidate class and the unassigned OTUs; ^b^The total refers to the total No. of the unaffiliated OTUs and the affiliated OTUs revealed by five primer sets for one sponge species; ^c^The number in brackets refers to the No. of the shared class level OTUs among four sponge species; ^d^The number in the brackets refers to the No. of the shared class level OTUs revealed by five primer set.

**Figure 2:**
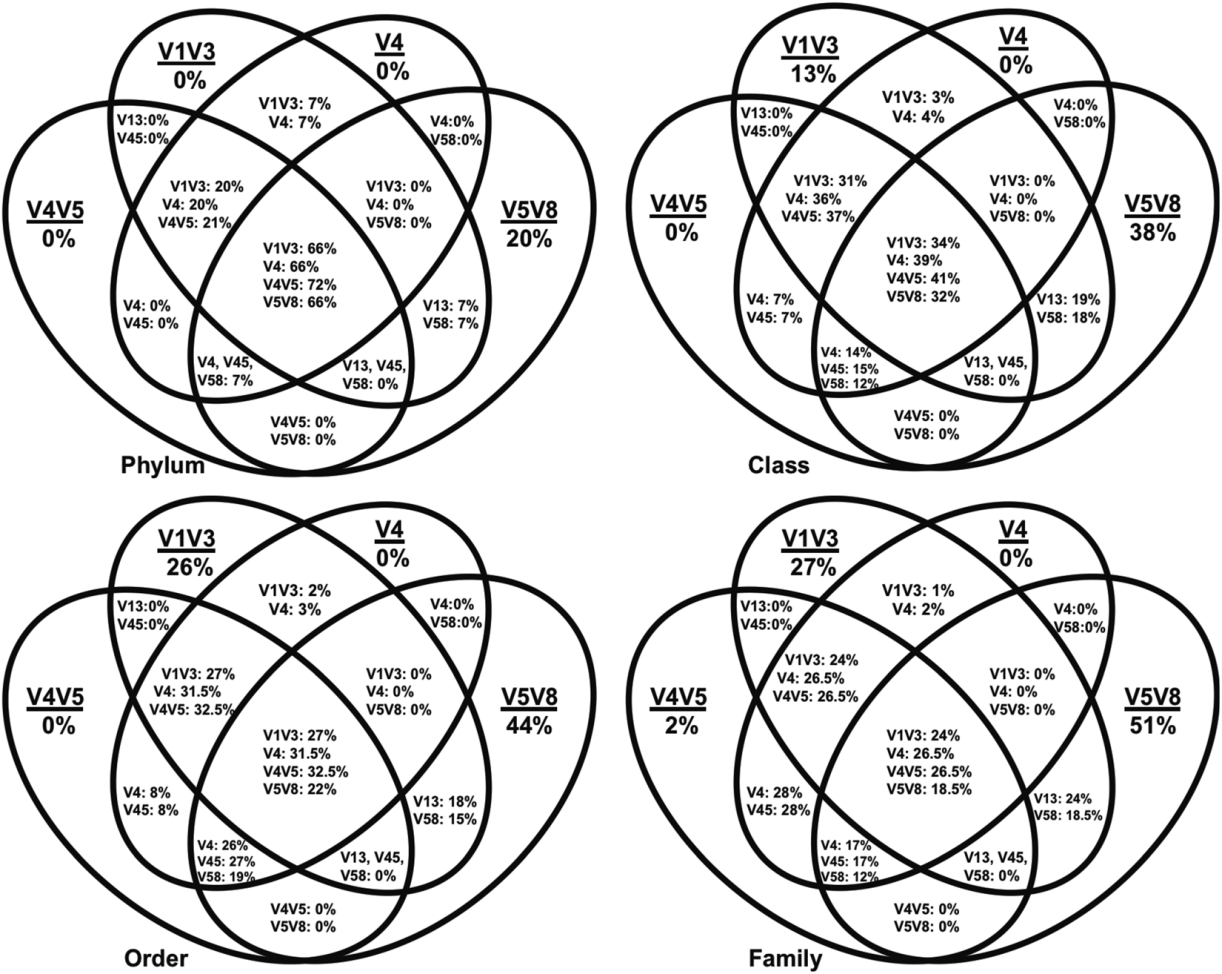
Distribution of affiliated (known & named) OTUs revealed by four region-specific primer sets V1V3, V4, V4V5 and V5V8 on Illumina MiSeq platform for the same sponge samples. The comparison is based on phylum, class, order and family levels of the microbial communities. The proportion % in the interactions of the Venn diagram = the number of the shared taxa/the total number of the taxa revealed by each of the primer sets. Each circle represents the whole bacterial OTUs revealed by one region-specific primer set with a sum of 100%.

**Figure 3:**
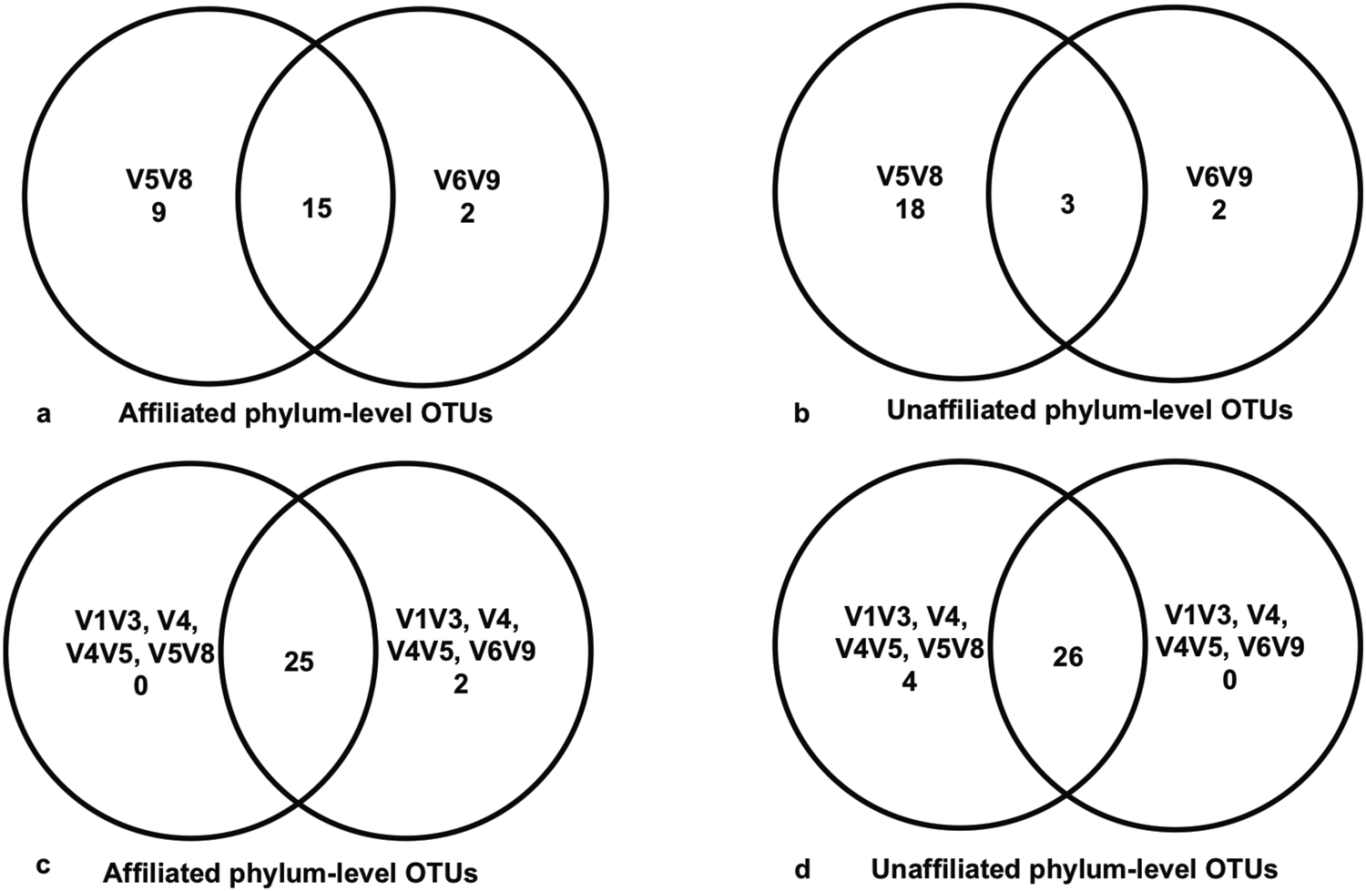
Comparison of the affiliated and unaffiliated phylum-level OTUs revealed by different region-specific primer sets on Illumina MiSeq platform for the same sponge samples. (**a**) Comparison between the datasets generated from primer sets targeting V5V8 and V6V9 for affiliated phylum-level OTUs; (**b**) Comparison between the datasets generated from primer sets targeting V5V8 and V6V9 for unaffiliated phylum-level OTUs; (**c**) Comparison between the regions V1V3, V4, V4V5, and V5V8 combined dataset and the regions V1V3, V4, V4V5, and V6V9 combined dataset for affiliated phylum-level OTUs; (**d**) Comparison between the regions V1V3, V4, V4V5, and V5V8 combined dataset and the regions V1V3, V4, V4V5, and V6V9 combined dataset for unaffiliated phylum-level OTUs.

The percentages of shared affiliated OTUs found in all four sponge samples, as inferred by four primer sets (Fig. 2; excluding V6V9), were 66–72%, 32–41%, 22–32.5% and 18.5–26.5%, at phylum, class, order and family levels, respectively. Again, the V5V8 dataset had the highest number of unique OTUs at all the four taxonomic levels beyond genus, not revealed by any other primer sets used. Compared to the performance of V6V9, seven more affiliated and 16 more unaffiliated phylum-level OTUs were obtained from the V5V8 dataset (Fig. 3a,b). When replacing V6V9 with V5V8 in the combined dataset (V1V3, V4, V4V5, and V5V8), two more affiliated phyla were detected but four unaffiliated phyla were not (Fig. 3c,d).

### Significant increase in revealing unaffiliated OTUs

The commonly used single V4 primer set revealed 26 phylum-level and 86 class-level unaffiliated OTUs from four sponge individuals representing four species (Tables 1 and 2). In contrast, 30 phylum-level and 103 class-level unaffiliated OTUs were obtained when combining the five primer sets (Tables 1 and 2). At phylum level, 48.5%, 51%, 45% and 37.1% of the OTUs jointly identified by the five primers were unaffiliated for each sponge *A*. *archeri*, *H*. *okadai*, *I*. *notabilis*, and *T*. *tubulifera*, respectively. Compared to the best primer for each of the four sponge species, an increase of 33.3%, 8.3%, 50%, and 18.2% of unaffiliated OTUs were observed, respectively, when using the combined dataset (Table 1). At class level, 57.3%, 59.1%, 53.6% and 47.7% OTUs were identified to be unaffiliated, with 45.7%, 6.6%, 44.4%, and 16.7% more compared to the best single primer set for *A*. *archeri* (V5V8), *H*. *okadai* (V4), *I*. *notabilis* (V4) and *T*. *tubulifera* (V4), respectively (Table 2). Overall, the combined dataset of the four sponges revealed 52.6% and 61.3% of unaffiliated OTUs at the phylum and class levels, respectively, compared to 45.6% and 51.2% when using the best single primer set.

Compared to the V4 dataset, the combined dataset revealed additional 13 phylum-level OTUs and 44 class-level OTUs (affiliated and unaffiliated OTUs) that represent 29.5% and 35.5% missed-out OTUs at the phylum and class levels, respectively, with the widely used primer set. It is interesting to note that the V5V8 region was the best in revealing affiliated OTUs for the four sponge species studied, while V4 performed the best in revealing unaffiliated OTUs for three of the four sponge species, except *A*. *archeri* (Tables 1 and 2). In addition, the shared OTUs at phylum level generated by the five primer sets constituted 33%, 20%, 25% and 29% of the total for *A*. *archeri*, *H*. *okadai*, *I*. *notabilis* and *T*. *tubulifera*, respectively. At class level, the shared OTUs were expected to be lower with 12%, 16%, 21% and 11% for *A*. *archeri*, *H*. *okadai*, *I*. *notabilis* and *T*. *tubulifera*, respectively.

### Significant improvement in sequence coverage

The total sequence reads generated from each of the five primer sets are shown in Table 3. The combination of the selected five primer sets revealed an increased microbial sequence coverage by 94%-550% (varying with different sponge species) in comparison to the single primer set V4, and by 90%-1075% when compared to V4V5 (Table 3). A high percentage (62%) of the bacterial and archaeal OTUs at phylum level that were reported as ‘likely missed OTUs’ by the V4 primer set (515F-806R)^23^ could be recovered by applying multiple primer sets to the four sponge species in this study (Tables 4 and S2).

**Table 3:** Increased sequences (%) of combined data using five region-specific primer sets based on the numbers of the sequence reads.

	V1V3	V4	V4V5	V5V8	V6V9	Combination^a^	Minimum increased %	Maximum increased %	Increased % compared with V4	Increased % compared with V4V5
*Aplysina archeri*	96338	100584	172013	298920	57184	327208	9.5	472.2	225.3	90.2
*Halichondria okadai*	69573	100280	55452	641381	47892	651704	1.6	1260.8	549.9	1075.3
*Igernella notabilis*	80920	100166	96798	162256	66088	194240	19.7	193.9	93.9	100.7
*Tedania tubulifera*	49392	42069	44587	129829	46002	162805	25.4	287.0	287.0	265.1

^a^The data in combination is calculated based on the total number of the phylum-level OTUs after removing the repeated ones. The sequence numbers of the OTUs shared among any two, three, four or all the five datasets generated from five primer sets are normalized following the method shown in section Materials and Methods - Sequencing throughput of five primer sets.

**Table 4:** The phylum-level OTUs (likely missed by the widely used 16S rRNA gene V4 region primer set 515F-806R) revealed by the combination of the primers specific to regions V1V3, V4, V4V5, V5V8, and V6V9. The likely missed phylum-level OTU list refer to the study of ^23^.

Phylum-level OTUs	V1V3	V4	V4V5	V5V8	V6V9
k__Archaea;p__Crenarchaeota	−	+	+	+	−
k__Archaea;p__Euryarchaeota	−	+	−	+	+
k__Bacteria;p__Acidobacteria	+	+	+	+	+
k__Bacteria;p__Actinobacteria	+	+	+	+	+
k__Bacteria;p__Armatimonadetes	+	+	+	−	−
k__Bacteria;p__Aquificae	−	−	−	−	+
k__Bacteria;p__Bacteroidetes	+	+	+	+	+
k__Bacteria;p__BRC1	−	+	−	−	+
k__Bacteria;p__Chlamydiae	−	+	+	+	+
k__Bacteria;p__Chlorobi	−	+	+	+	+
k__Bacteria;p__Chloroflexi	+	+	+	+	+
k__Bacteria;p__Cyanobacteria	+	+	+	+	+
k__Bacteria;p__Firmicutes	+	+	+	+	+
k__Bacteria;p__Fusobacteria	+	+	+	+	−
k__Bacteria;p__Gemmatimonadetes	−	−	−	+	+
k__Bacteria;p__GN02	+	−	−	+	−
k__Bacteria;p__Lentisphaerae	+	+	+	+	−
k__Bacteria;p__Nitrospirae	+	+	+	+	−
k__Bacteria;p__OD1	+	+	+	+	+
k__Bacteria;p__OP11	+	+	+	+	−
k__Bacteria;p__OP3	−	+	+	+	−
k__Bacteria;p__Planctomycetes	+	+	+	+	+
k__Bacteria;p__Proteobacteria	+	+	+	+	+
k__Bacteria;p__Spirochaetes	+	+	+	+	+
k__Bacteria;p__SR1	−	+	−	−	−
k__Bacteria;p__Synergistetes	+	−	−	−	−
k__Bacteria;p__Tenericutes	−	+	+	+	−
k__Bacteria;p__Thermotogae	−	−	−	−	+
k__Bacteria;p__TM7	+	+	+	+	+
k__Bacteria;p__Verrucomicrobia	+	+	+	+	+
k__Bacteria;p__WS3	−	−	−	+	+
k__Bacteria;p__WWE1	−	−	−	+	−

### High resolution on low abundant microbial taxa detection

The detection limit preventing the discovery of low abundant microbial taxa associated with sponges was investigated and addressed. As a result, 29 candidate phylum-level OTUs were revealed from only 2.3% sequences in total (Tables 1 and S3). Each of the candidate OTUs, on average, contributing 0.08% were considered to be the low abundant taxa, when compared to a much higher number of the sequences (87.62%) for a similar number of the affiliated phylum-level OTUs. In addition, the ‘Unassigned phylum-level OTU’ generated from the data processing was grouped as only one unit but made up 10.8% of total sequences. It covered all the sequences that could not match any of the entries in the database, so could not be confirmed or identified to known and candidate phyla. It would be reasonable to expect that these sequences contribute to a large number of novel microbial phyla when the unknown OTUs were rare and low-abundant (<0.1%). Archaea showed low abundance at both phylum and class levels of the sponge microbiomes studied (Supplementary Table S2). All the primer sets except the one specific to region V4 revealed archaeal OTUs that all belong to affiliated OTUs with relative sequence abundance of 0.01–1.35%.

### Microbiome comparison by different primer sets

**Figure 4:**
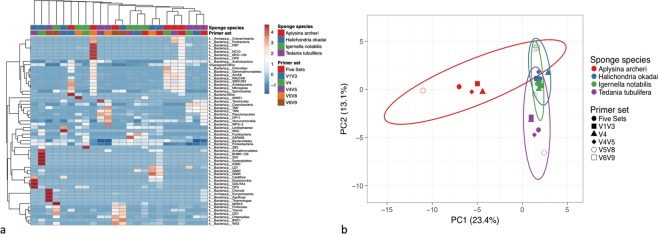
Microbial community taxonomic heatmap and Principal Component Analysis based on phylum-level microbial OTUs generated by five primer sets and combined dataset. (**a**) Microbial community heatmap clustered using correlation distance and average linkages; (**b**) Principal Component Analysis, unit variance scaling for each row in the data matrix and singular value decomposition for missing data imputation when calculating the principal components.

The comparison among the microbial communities of four sponge species was indicated in a heatmap based on the phylum-level microbial OTUs generated by different primer sets (Fig. 4a). The microbial communities of each sponge species revealed by different single primer sets showed unmatched clustering patterns. Sponges *T*. *tubulifera*, *A*. *archeri*, and *I*. *notabilis* showed clustered microbial community profiles that were revealed by only two single primer sets and the combined dataset. Using combined dataset, sponge *T*. *tubulifera and A*. *archeri* have more similar microbiomes to *H*. *okadai*. In contract, using single primer set V4, the microbial communities of sponge *T*. *tubulifera and A*. *archeri* showed higher similarity to that of *I*. *notabilis*. The variations among the five primer sets when revealing the same sponge species are shown in Fig. 4b. Sponge *A*. *archeri* had the largest divergence of the microbiomes generated by different primer sets.

## Discussion

A combination of five region-specific primers covering all the nine variable regions of the 16S rRNA gene was applied to characterize the microbiome of four sponge samples. Each region-specific primer set showed different capacities to profile different parts of the microbiome when analyzing affiliated OTUs (Fig. 2). Individually, none of them had adequate coverage of the microbiome to be a truly ‘universal’ primer set for microbiome studies.

A more complete OTU coverage of sponge microbiomes was obtained by jointly analyzing the different variable regions of the 16S rRNA gene. Each of the five primer sets contributed substantially different parts to the combined unaffiliated microbial profiles at the phylum and class levels (Tables 1 and 2). The combined dataset achieved a coverage superior to that of any single region-specific primer set. It uncovered 29.5% and 35.5% of phylum-level OTUs and class-level OTUs, respectively, that are missed by the most commonly used primer set targeting the V4 region (Tables 1 and 2). In relation to the sequence coverage compared to this commonly used primer alone, an increase of 0.9 to 5.5-fold was found for the four sponge species (Table 3). The results prove that a ‘universal’ single primer set is far from adequate and the arguments made on grounds of cost or ease of data analysis cannot be sustained or justified in the face of this scientific evidence that reveals more comprehensive microbiomes. Moreover, the incomplete data could result a misleading investigation of the microbial communities due to the highly divergent profiles generated from different primer sets (Fig. 4).

An in silico PCR using the latest release SILVA SSU r132 (13 Dec. 2017)^24^ was further employed to evaluate the efficiency of the region-specific primer sets on revealing microbiota at different taxonomic ranks^25^. Different microbiota coverages from rank domain to species were obtained (Supplementary Datasets S3–S7, for the primer sets targeting regions V1V3, V4, V4V5, V5V8, and V6V9, respectively). Primer set for regions V6V9 had a much lower microbiota coverage compared to other four primer sets (Supplementary Table S5), which was also reflected in the comparison about OTU number and sequence reads among the primer sets to reveal microbiota of four sponge species (Tables 1–3). Primer sets for regions V4V5 and V5V8 performed comparable efficiency, and better than primer sets for regions V1V3 and V4 (Supplementary Table S5). For amplicon-based sequencing, the taxonomy classification resolution of each single primer set could be, to a larger degree, impacted by its inadequate sequence entries in the database. To amplify the full length of the 16S rRNA gene using multiple-primer combinations could efficiently make use of the different preferences of each single region in revealing the microbiome. This multi-primer approach can significantly increase the microbiota coverage and also greatly improve the resolution of the taxonomic classification, thought there is still a limitation on the identification at the species level.

Other amplicon-based metagenomic analyses of marine sponges are also considered for a parallel comparison. The Sponge Microbiome Project (SMP)^1^ globally surveyed 804 sponge specimens belonging to 81 sponge species, and identified only 41 phylum level OTUs (16 unaffiliated and 25 affiliated) (Supplementary Table S4). By comparison, in our study 14 more unaffiliated and 2 more affiliated phylum-level OTUs were revealed from only four sponge species. This translates to a huge opportunity to discover new microbial lineages from sponges that may contribute to revealing the said taxonomic ‘blind spots’. These findings highlight a potential reservoir of untapped marine microbial resources. Different region-specific primer sets showed various efficiencies on uncovering the microbiota, but always with less capacity than a 16S rRNA gene full-length amplicon by multi-primer approach. A recent study analyzed 19 sponge species by region V4 following the protocol in SMP and only uncovered 18 bacterial phyla/candidate phyla in total^26^. Using the same region-specific primer set for V5V8 as our study, Fieth *et al*.^27^ analyzed the ontogenetic changes in the bacterial symbiont community of the sponge *Amphimedon queenslandica* and revealed 14 phyla/candidate phyla that is much less than what we found in the current study with a minimum 33 phyla/candidate phyla for one sponge species (Table 1). Archaea is generally considered to be low abundant in marine sponges, using the primer set targeting regions V5V6 revealed that the microbiome of sponge *Inflatella pellicula* was dominated by archaeal sequencing reads with a single archaeal OTU^28^. However, a much lower number of bacterial phyla (22 phyla, including candidate ones) were revealed compared to our results with at least 33 bacterial phyla from one sponge species. Similarly, using single primer set based approach for regions V1V2 and V6V8, core microbiome stability of sponges was demonstrated with the geographical and temporal variations^29,30^, but only with 11 phyla and six phyla, respectively. When combining two 16S rRNA gene regions of V3 and V6, *Asbestopluma hypogea*-associated microbiome was assigned to 20 bacterial and two archaeal phyla (45 classes in total)^31^ that is still with significantly smaller microbial coverage than the findings in the current study (minimum 33 phyla and 88 classes) (Tables 1 and 2). Therefore, each of the 16S rRNA gene regions is essential to reveal a comprehensive sponge microbiome profile. In addition, combining variable regions of the 16S rRNA gene has proven to allow more OTUs to be assigned to a certain taxonomic affiliation by a study using mock samples^32^, which theoretically explained why a 1.5- to 4-fold increase in genus level classification was obtained in our study using the combined data from five primer sets when compared to any single pair of primers (Fig. 1).

To illustrate the advantages and limitations of the amplicon based and metagenomic sequencing, a recent study^23^ aligned small-subunit ribosomal RNA (SSU rRNA) gene sequences recovered from metagenomes (38,454 SSU rRNA) and genomes (25,439 SSU rRNA), which cover 26 sample types in four categories - terrestrial, aquatic, host-associated, and engineered, with commonly used PCR primer sets (V4: 515F-806R and V4V5: 515F-926R from the Earth Microbiome Project^14^; V3V5: 341F-785R and 357F-926R from the Human Microbiome Project^15^). Approximately 10% of environmental microbial sequences were reported to be missed from classical PCR-based SSU rRNA gene surveys, mainly including the candidate phyla and as yet uncharacterized Archaea^23^. Combining any two primer pairs could not significantly improve taxonomic coverage. Even when the four primer sets were combined, 5.5% of sequences could not be detected from metagenomic data, which seems to imply that the use of multiple primer sets only slightly improves the recovery^23^. Notably, these four primer sets applied in their study are limited to the V3-V5 regions which is less than half of the full length of the 16S rRNA gene. In contrast, when more variable regions covering the full length of the 16S rRNA gene were sequenced, a significant improvement of the taxonomic coverage was achieved, as shown in our study to reveal a more comprehensive sponge microbiome profile (Tables 1–4; Fig. 2), especially for unaffiliated taxa, including both candidate and unassigned taxa.

Moreover, the metagenome of the sponge *Aplysina aerophoba*^33^, which belongs to the same genus as *A*. *archeri* in our study, is introduced here to provide a comparison between these two techniques. Sponge *A*. *aerophoba* metagenome was presented by hybrid assemblies derived from Illumina short-read and the PacBio long-read data with subsequent un-targeted differential coverage binning^33^. In total 13 bacterial phyla (11 affiliated and 2 candidate phyla) were identified. Compared to this metagenomic data, a substantial increase in the OTU coverage was obtained in our study with 20 more phyla (5 affiliated, 14 candidate, and one unassigned phylum) revealed, though the dissimilarity between the host species within the genus and the different sampling locations could impact the associated microbiomes. Additionally, the V4 region was also sequenced by Illumina MiSeq in the same study^33^ which revealed 26 bacterial phyla, including 19 affiliated (one archaeal phylum), 6 candidate, and one unassigned phylum. In comparison, our study revealed 10 more candidate phyla and one more archaeal phylum, though with three less affiliated phyla (Table 1). The data generated from the improved multi-primer approach here can be considered to suggest the amplicon-based sequencing has not reached its saturation. The well-validated protocols will significantly reduce the biases due to regions/primers choices and provide a more comprehensive microbial profile of any given environmental samples.

In fact, the amplicon-based approach could be more efficient and more targeted than metagenomic sequencing to identify the microbial taxa when conducting low-microbial biomass and highly host-contaminated samples (e.g. sponges)^34^. Contamination from host-derived DNA and organelles may obscure microbial signatures and population-averaged microbial genomes tend to be inaccurate owing to assembly artefacts^34^. In addition, compared to the abundant 16S rRNA gene data in the database, the metagenome entries limit the coverage of a microbial profile. For the members of a microbiome not yet characterized by culture-based methods, a fraction of reads in the metagenome may still remain unmatched after assembly, and the size of this fraction is highly dependent on community structure and complexity^35^.

A more reliable and comprehensive diversity profile has been achieved for the structure and composition of microbial communities of different sponges (Supplementary Fig. S4). Based on examination by Transmission Electron Microscopy, *A*. *archeri* was identified as a High Microbial Abundant (HMA) sponge^36^; the sponge genera *Halichondria*^37^ and *Tedania*^36^ were classified as Low Microbial Abundant (LMA) sponges. The four selected species in our study were therefore considered to represent both HMA and LMA sponges. Interestingly, the two LMA sponges (*H*. *okadai* and *T*. *tubulifera*) were found to show higher numbers of the OTUs than the HMA sponge (*A*. *archeri*) (Tables 1 and 2). It could imply that all the 16S rRNA gene regions accompanied with a well validated protocol are essential to profile the sponge microbiomes.

Many published studies have reported a profound impact of targeting different variable regions of the 16S rRNA gene on the microbial diversity difference (community richness and evenness)^9,10,16–18^. However, these differences are often referred to as ‘biases’, rather than truly different components of the microbiome revealed by sequencing different variable regions. It is fundamentally critical to recognise that sequencing a combination of different regions could complement the ability of each region to add to the completeness of the microbiome as the comparison and match of sequences in one region sequenced does not exclude the possibilities of unmatched sequences in the other eight regions. The method proposed here can be applied effectively to provide a more complete sponge microbiome. Having a complete microbiome will provide an essential foundation to uncover the vast unknown sponge microbiota and explore their roles and functions in the marine sponge holobionts.

## Methods

### Experimental design

The experimental flowchart is shown in Supplementary Fig. S5. It was designed with selection and collection of representative sponge specimens, followed by DNA extraction, PCR amplification, sequencing, and data processing. Biological and technical replicates were applied as indicated. The sequence information generated by the technical and biological replicates were only analyzed separately in terms of the sequence reads summary and beta-diversity analysis for the purpose to validate the consistency and reliability of this proposed approach. To achieve an enhanced microbiome coverage, the datasets generated from the five primer sets were merged following a rational protocol to reveal the community of each sponge species as described in a later section. The substantially different performances of the five primer sets targeting different 16S rRNA gene regions were highlighted by analyzing and presenting the datasets generated from different primers sets separately.

To better compare with the literature, the data were analyzed at phylum level or class level, in particular for distinguishing the classes in the phylum Proteobacteria, to demonstrate the improved microbial coverage of the multi-primer approach. The different performances among different primer sets were illustrated at phylum level. The analyses for the microbiota at lower taxonomic levels (order, family, and genus) were only presented for affiliated OTUs (known OTUs) due to the limited value when most of the OTUs are unclassified at these ranks. Affiliated and unaffiliated OTUs were separately presented to investigate the advantage of the proposed multi-primer approach.

### Sponge collection and community DNA extraction

Sponge specimens were collected via scuba diving at depths of 4–15 metres at Rapid Bay, Adelaide, South Australia (35°31'16.6“S, 138°11'07.5“E) in February and March of 2013. Each specimen was kept separately using a sterile plastic bag and placed in an ice box during transportation. Specimens were flushed with 0.22 µm membrane filtered seawater to remove loosely attached microbes and debris. About 10 cm^3^ sponge tissue specimens were cut into small pieces with a sterile blade and stored at −80 °C for subsequent DNA extraction. The specimens were identified according to their molecular classification in conjunction with the morphological characterization following our previously developed protocol^30^. They are *Aplysina archeri* with accession number KJ620395^38^, *Halichondria okadai* - KJ801656^38^, *Igernella notabilis* – KJ620376^38^, and *Tedania tubulifera -* KJ620377^38^, which belong to four different orders Verongida, Suberitida, Poecilosclerida, and Dendroceratida, respectively.

DNA extraction utilized in this study was the CTAB-based method^39,40^. Briefly, the frozen sponge tissues were freeze-dried in −80 °C for 4–12 hours depending on the tissue size. The dried tissues were ground and suspended in the sterile distilled water for one hour, and after the low speed (600 × g) centrifugation the tissue deposited at the bottom of the tube was collected. The CTAB extraction buffer was applied to lyse tissues, which was combined with polyvinylpyrrolidone (PVP) and β-mercaptoethanol to help remove phenolic compounds and tannins in the extract. A bead-beating step using 1.0 mm diameter silica beads (Biospec Products) was applied to increase the DNA release^41^. The purified DNA was resuspended in 35 μl of sterile distilled water. Purity and quantity of DNA were determined with a NanoDrop ND-1000 Spectrophotometer (Thermo Scientific, Wilmington, DE, USA). At least eight specimens of each sponge species were used for DNA extraction. Multiple extractions (n ≥ 3) were carried out for each specimen to achieve sufficient DNA yields. Three qualified DNA samples (A260/280: 1.8–2.0; Conc. > 100 ng/µl) extracted from different sponge individuals for each species were selected and kept at −20 °C for subsequent PCR reactions and sequencing.

### Illumina MiSeq amplicon library and sequencing

Five primer sets V1V3^42^, V4^43^, V4V5^44^, V5V8^17^, and V6V9^44,45^ targeting all nine hyper-variable regions were selected for Illumina sequencing in order to cover the full-length of 16S rRNA gene (primer details in Supplementary Table S6 and the targeted locations in Supplementary Fig. S6). PCR was performed based on the protocol presented in^46^. Briefly, both of the forward and reverse primers were added to the 5′ and 3′ Illumina adapter, respectively. The PCR reaction conditions were 2 mM MgCl_2_, 0.2 µM each primer and 200 µM dNTPs. The PCR conditions were 94 °C for 3 mins, followed by 35 cycles of 94 °C for 45 s, 50 °C for 60 s, 72 °C for 90 s, and a final elongation step at 72 °C for 10 mins. For each primer set, negative controls and triplicate amplifications were applied for each of the three qualified DNA samples extracted from different individuals belonging to the same sponge species. After the purification and quantification, one of the three amplicons (A260/280: 1.8–2.0) with the highest yield was selected. The experiments for all the five primer sets follow the same protocol to obtain, in total, 15 amplicons per sponge species for Illumina sequencing.

The amplicons derived from different individuals of the same sponge species were sequenced multiple times (n ≥ 3) on an Illumina MiSeq Sequencer from both ends of paired-end library preparations (2 × 300 bps), using sequencing kit version 3.0 followed by base-calling using Illumina Genome Analyzer Pipeline software (GAPipeline version 1.4.0.) with default parameters. The DNA standard provided by ZymoBIOMICS™ was utilized to firstly evaluate the microbiota recovery efficiency of different primer sets and the multi-primer-combined approach, and then was employed as the quality control (both Cellular Standard and DNA Standard), including genera *Bacillus*, *Enterococcus*, *Escherichia*, *Lactobacillus*, *Listeria*, *Pseudomonas*, *Salmonella*, and *Staphylococcus*. The quality control was utilized in each run of multiple sequencing to validate the measured microbial composition at species level against the theoretical composition of the Microbial Community Standard. The highest throughput of the sequencing reads among the replicates (technical and biological replicates) belonging to the same sponge species generated by one primer set was selected to reveal the microbial community, not only because the combination of the data with highest throughput generated from each of the five primer sets could reflect the best capacity of the proposed multi-primer approach in terms of the sequence reads but also to streamline the feasibility and practicality of the protocol. The selected throughputs from each of the five primer sets were employed together to obtain significantly increased sequencing reads so as to profile community more comprehensively.

### 16S rRNA sequencing data processing

#### Demultiplexing and dataset quality filtering

Before processing 16S amplicon sequencing data, we evaluated two most widely adopted pipelines, the quantitative insights into microbial ecology (QIIME) (version 1.9.1)^47^ and mothur^48^, with default parameters. The DNA standard (ZymoBIOMICS™) mentioned above was employed as the input Microbial Community Standard. At the genus level, these two pipelines offered similar results, but QIIME showed higher Pearson correlation coefficient between the expected (input) and obtained relative abundance. Therefore, QIIME was applied for our subsequent analyses in this study.

The demultiplexing and quality control for the Illumina MiSeq dataset was processed by script *split_libraries*.*py* in QIIME pipeline (version 1.9.1)^47^: The multiplexed reads were assigned to samples based on their nucleotide barcode (demultiplexing). Quality filtering was performed based on the characteristics of each sequence, removing any low quality or ambiguous reads. Briefly, the first 20 bases of all fastq files were trimmed to remove the primer sequence, and quality trimmed to remove poor quality sequence using a sliding window of 4 bases with an average base quality above 15 using the software Trimmomatic (Version 0.35)^49^. Chimeric sequences were detected and removed by the USEARCH tool^50^. Based on the locations of the primer sets, there are 491 bp, 291 bp, and 408 bp amplicons analyzed for regions V1V3, V4, and V4V5, respectively. For the three types of amplicons, the R1 and R2 reads were individually trimmed to 250 bases and then were merged into one contig based on their consensus sequence. In terms of the amplicons of V5V8 (589 bp) and V6V9 (566 bp), the taxonomy assignment was conducted using R1 or R2. We found R1 typically performed higher quality than R2, accordingly, R1 was selected for the analysis of amplicons V5V8 and V6V9^51,52^. The generated fastq format files were finally converted to fasta format files (seqs.fna) for next step analysis.

#### Closed-based OTU picking

Considering the pros and cons of the three high-level protocols for OTU picking in QIIME pipeline (de novo, closed-reference, and open-reference OTU picking), closed-reference picking was selected in our study due to its faster speed and better taxonomy (see OTU picking strategies in QIIME^47^). Briefly, reads were assigned to OTUs using a closed-reference OTU picking protocol^47^. The output file (seqs.fna) from the script *split_libraries*.*py* was then used as the input file to run the following workflow (*pick_closed_reference_otus*.*py –i seqs*.*fna -r refseqs*.*fna -o otus_w_tax -t taxa*.*txt*). Typically, reads were assigned to OTUs based on their best hit to Greengenes database (version: gg_13_8_otus/rep_set/97_otus.fasta)^53^ at greater than or equal to 97% sequence identity^54^. Greengenes database was applied as references in this study due to its intermediate performance on the taxonomies at each rank from phylum to genus compared to SILVA and RDP^55^. In closed-reference OTU picking process, any reads which did not hit a sequence in the reference sequence collection were excluded from downstream analyses^56^. An OTU map (otu_map.txt) was generated as the output. A representative sequence (the OTU centroid sequence) for each OTU was then picked. This representative sequence was used for taxonomic identification of the OTU and phylogenetic alignment. Next, taxonomy was assigned to each representative sequence. By default, QIIME applied the uclust^50^ consensus taxonomy classifier to attempt to assign taxonomy to each representative sequence. The full lineage information for each sequence (tax_assignments.txt) was one of the output files. Then align the representative sequences of each OTU using PyNAST tool^57^ and filter the alignment to remove all gaps and the sequences known to be excessively variable. The filtered alignment file was then used to build a phylogenetic tree using a tree-building program (e.g. FastTree^58^). Finally, using taxonomic assignments (tax_assignments.txt) and the OTU map (otu_map.txt), QIIME assembled an OTU table (otu_table.biom). The table summarized the OTU abundances in each sample with taxonomic identifiers for each OTU.

#### Affiliated OTUs revealed by five primer sets

OTUs were grouped according to different taxonomic levels (phylum, class, family, and genus) with the workflow script (*summarize_taxa_through_plots*.*py -i otu_table*.*biom -o taxa_summary -m Fasting_Map*.*txt*). The script generated new tables (txt format files) at all the taxonomic levels. Each taxonomy table contains the relative abundances of taxa within each sample, which were applied for the downstream analyses.

Affiliated OTUs are the ones assigned to known identities with certain taxonomic classifications. The unique and the total number of genus-level OTUs generated by five primer sets for regions V1V3, V4, V4V5, V5V8, and V6V9 were calculated and compared within a sponge species aiming to specifically indicate whether different primer sets targeting different variable regions reveal vastly different parts of a given microbial community. The unique OTUs are defined as the ones can only be revealed by one primer set. The total affiliated OTU number was obtained by counting the unique OTUs generated by each of the five primer sets together and excluding the number of repeated ones. Importantly, we have taken into account that different variable regions have different resolutions of certain taxa, hence a given sequence can be unambiguously assigned to a certain genus using one variable region but not another. Since we only considered the affiliated taxa, we produced a conservative evaluation when five primers were combined. To further demonstrate their various performance on profiling sponge microbiomes, the datasets of four sponge species selected in the study were combined to analyze the distribution of the microbial OTUs (from phylum to family level) revealed by five primer sets.

#### Unaffiliated OTUs revealed by five primer sets

The unaffiliated OTUs are the ones assigned to unapproved taxonomic identities, including Candidate OTUs and Unassigned OTUs (counted as one OTU). The analyses were designed to validate if each single primer set for a particular region(s) shows significant limitations on profiling a complete microbial community, and also to validate whether applying multiple primer sets covering all nine variable regions of the 16S rRNA gene is a practical approach to respond to the limitations. The affiliated and unassigned OTUs (phylum and class levels) were analyzed separately based on different primer sets for each sponge species. A recent comprehensive sponge microbiome survey^1^ was compared with our study to illustrate the improved coverage of microbial taxa revealed by our proposed multi-primer approach.

#### Sequencing throughput of five primer sets

The sequencing throughput (number of sequence reads) was calculated to illustrate different capacity of the five primer sets and the improved performance of the multi-primer strategy compared to any single one primer set for each sponge species. The improved sequence throughput was predicted according to the maximum extent of sequence reads increase by considering all the OTUs revealed by five primer sets. Typically, the sequence reads of the unique OTUs generated by each of the five primer sets were added directly. In terms of the shared OTUs revealed by any two, three, or four primer sets and the ones detected by all five primer sets, the highest sequence read offered by different primer sets was considered to be the representative throughput for this particular OTU to illustrate the potential power of the multi-primer approach.

The relative abundance (%) of sequence reads for affiliated, candidate, and unassigned OTUs were analyzed separately based on different primer sets for each sponge species. It was designed to test whether the low abundant microbial taxa (<1%) and rare microbial taxa (e.g. Archaeal taxa) can be detected by this proposed approach, as well as to evaluate the performance of each single primer set and their combination, in particular, on exploring the untapped microbial OTUs (unaffiliated OTUs). Notably, the OTUs belonging to eukaryotes revealed by primer set V6V9 were excluded in this analysis, and the sequence relative abundance (%) was recalculated. Importantly, the combined data was evaluated by considering different contributions (sequence reads) of each primer set for the same sponge species or different proportions taken by four sponge species generated by the same primer set. Moreover, it was also designed to further demonstrate the improved OTUs coverage of the proposed multi-primer approach by comparing the microbial profile (phylum level) in our study with the likely missed OTUs reported in a recent published work^23^ that shows the amplicon-based microbiome study approach missed substantial OTUs when compared to the metagenomics method.

#### Microbiome analysis

The taxonomy metadata was further conducted by ClustVis to visualize the Principal Component Analysis (PCA) and microbial community heatmap^59^. Raw data were pre-processed under the parameters: maximum 99.99% of missing values in rows and columns; unit variance scaling for each row; and singular value decomposition for missing data imputation when calculating the principal components. The total variance of the principal components is explained in X and Y axis. In the heatmap, the rows are cantered, and the unit scaling is applied. Both rows and columns are clustered using correlation distance and average linkages.

### Accession codes

The raw sequencing reads were deposited in the GenBank at the National Center for Biotechnology Information (BioProject ID: PRJNA490791).

## Supplementary information

Supplementary Information

Dataset 1

Dataset 2

Dataset 3

Dataset 4

Dataset 5

Dataset 6

Dataset 7

## Data Availability

All data generated or analyzed during this study are included in this published article and its Supplementary Information files. The raw sequencing reads were deposited in GenBank at the National Center for Biotechnology Information (BioProject ID: PRJNA490791).
