# Supplementary material for: Uncovering the hidden marine sponge microbiome by applying a multi-primer approach
Source: Sci Rep. 2019 Apr 17;9:6214. doi: 10.1038/s41598-019-42694-w (PMC6470215; doi:10.1038/s41598-019-42694-w)
Supplement: Supplementary file 1 — Supplementary Information [file 41598_2019_42694_MOESM1_ESM.pdf]

# Uncovering the hidden marine sponge microbiome by applying a multi-primer approach

Qi Yang [1,2], Christopher M. M. Franco [1], Wei Zhang [1,2*]

[1]Centre for Marine Bioproducts Development, College of Medicine and Public Health, Flinders University, Adelaide, South Australia, 5042, Australia;

[2]Center for Marine Drugs, State Key Laboratory of Oncogene and Related Genes, Department of Pharmacy, Renji Hospital, School of Medicine, Shanghai Jiao Tong University, Shanghai 200127, China.

*** Corresponding author:**

Wei Zhang; wei.zhang@flinders.edu.au

# Supplementary Information

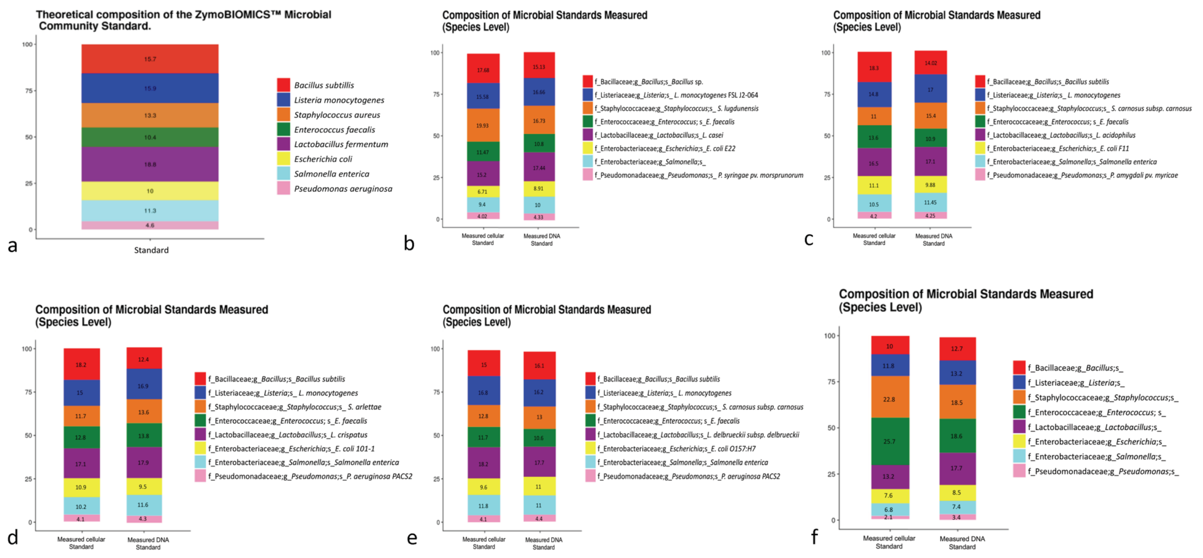


**Supplementary Fig. S1 Theoretical microbial composition and measured composition of the ZymoBIOMICS™ Microbial Community Standard employed as quality control in each sequencing run of the technical replicates.** a. Microbial Community Standard; b. Composition of Microbial Community Standard measured by V1V3 amplicon; c. Composition of Microbial Community Standard measured by V4 amplicon; d. Composition of Microbial Community Standard measured by V4V5 amplicon; e. Composition of Microbial Community Standard measured by V5V8 amplicon; f. Composition of Microbial Community Standard measured by V6V9 amplicon. Cellular Standard: bacterial cultures were mixed so that the community was comprised of equal numbers of cells; DNA Standard: DNA was extracted from pure bacterial cultures and then mixed so that the community was comprised of equal amounts of DNA.

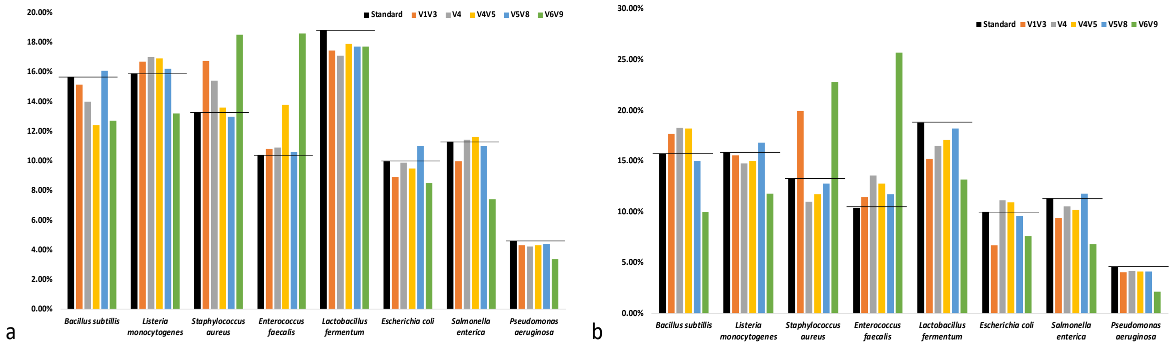


**Supplementary Fig. S2 Efficiency evaluation of the region-specific primer sets on revealing the microbial composition of the ZymoBIOMICS™ Microbial Community Standard.** a. Performance of the five primer sets for the mock microbial community of Cellular Standard; b. Performance of the five primer sets for the mock microbial community of DNA Standard. The black lines in the bar chart are added to denote the standard. Cellular Standard: bacterial cultures were mixed so that the community was comprised of equal numbers of cells; DNA Standard: DNA was extracted from pure bacterial cultures and then mixed so that the community was comprised of equal amounts of DNA.

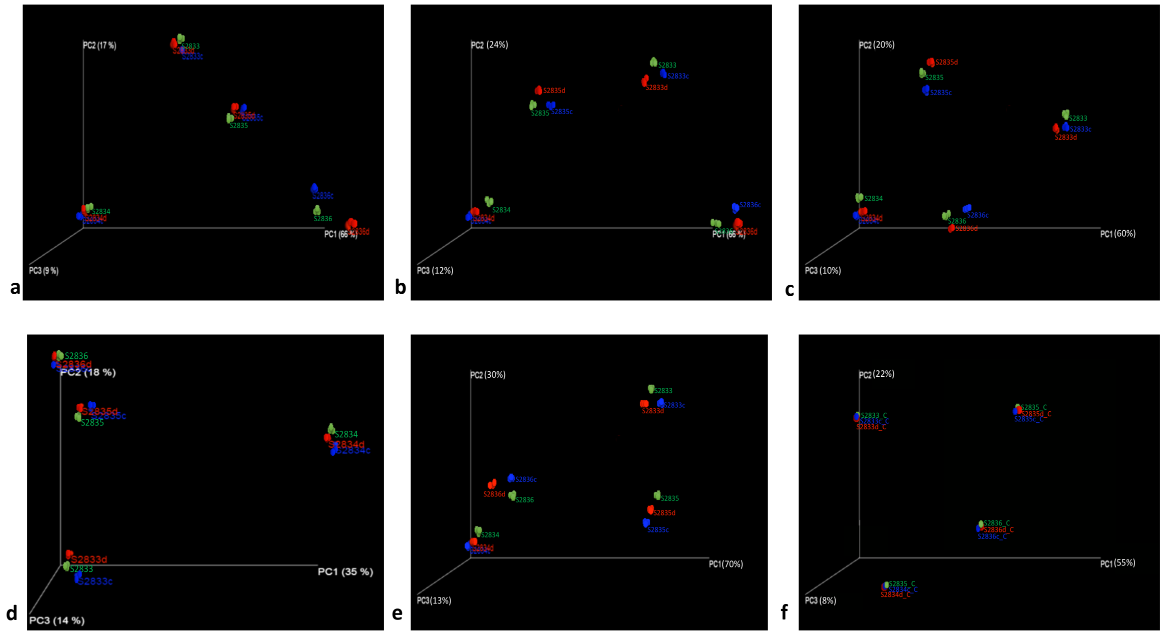


**Supplementary Fig. S3 Principal Coordinates Analysis (PCoA) plots showing the similarity of microbial communities of 12 sponge specimens revealed by five primer sets for 16S rRNA gene regions V1-V9 using weighted UniFrac metric of beta-diversity analysis. a.** regions V1V3; **b.** region V4; **c.** regions V4V5; **d.** regions V5V8; **e.** regions V6V9; **f.** five primer sets combined. S2833: *Aplysina archeri* (order: Verongiida), S2834: *Halichondria okadai* (order: Suberitida), S2835: *Igernella notabilis* (order: Dendroceratida), S2836: *Tedania tubulifera* (order: Poecilosclerida); Red, blue, and green dots with the same number represent three biological replicates belonging to same sponge species; small letter c and d distinguish the additional two biological replicates; for **a-e**, the multiple dots (≥3) with the same colour refer to the technical replicates; for **f**, capital letter _C means the Combined dataset.

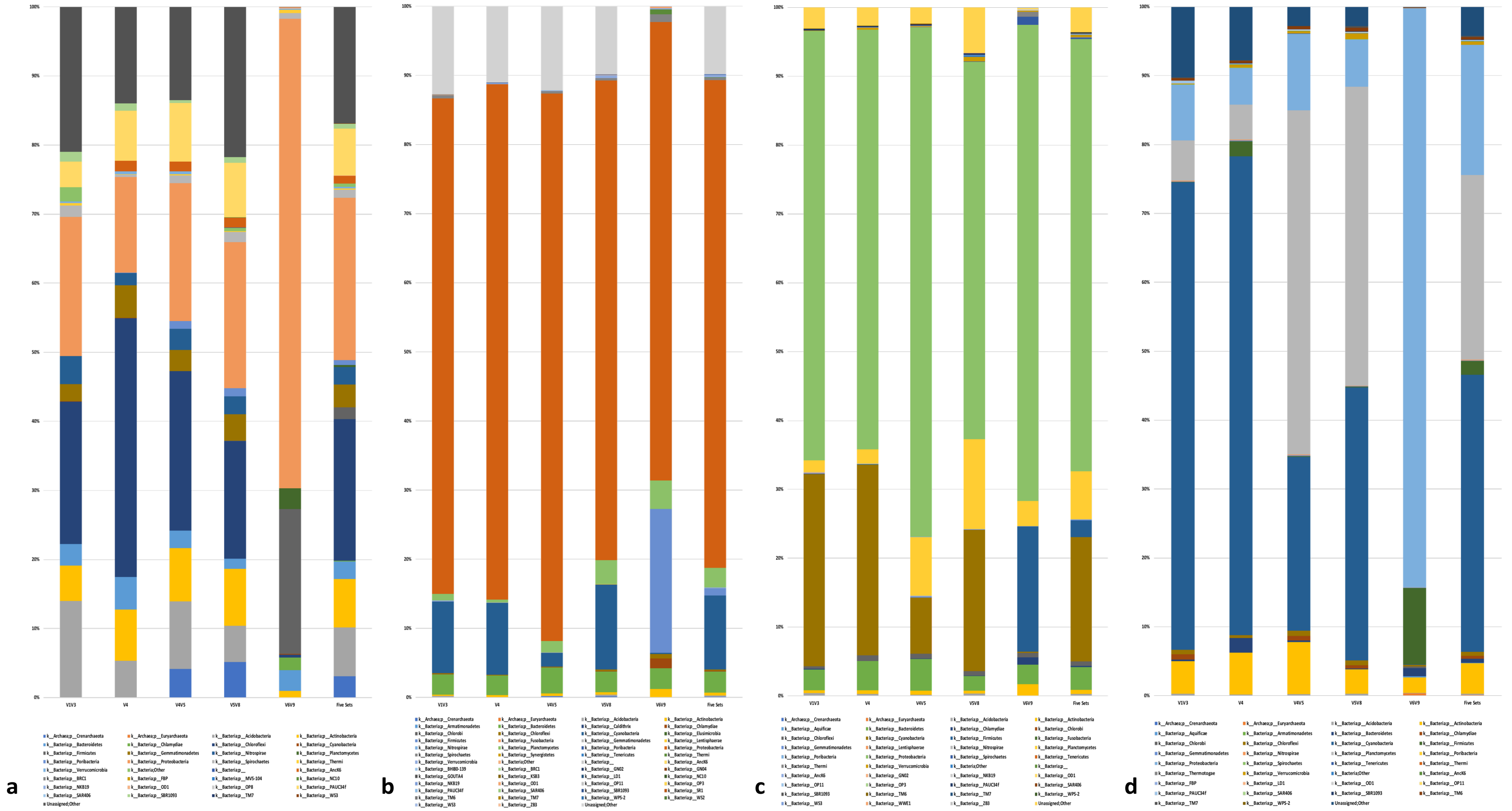

**Supplementary Fig. S4** Microbial community of four sponge species revealed by each region-specific primer set on Illumina MiSeq platform. The specific primers are for the V1V3, V4, V4V5, V5V8 and V6V9 regions of 16S rRNA gene. The microbial profile using all five primer sets for each sponge species was showed as the last column in each chart. a. Sponge *Aplysina archeri*; b. Sponge *Halichondria okadai*; c. Sponge *Igernella notabilis*; d. Sponge *Tedania tubulifera* (see Supplementary Dataset S2).

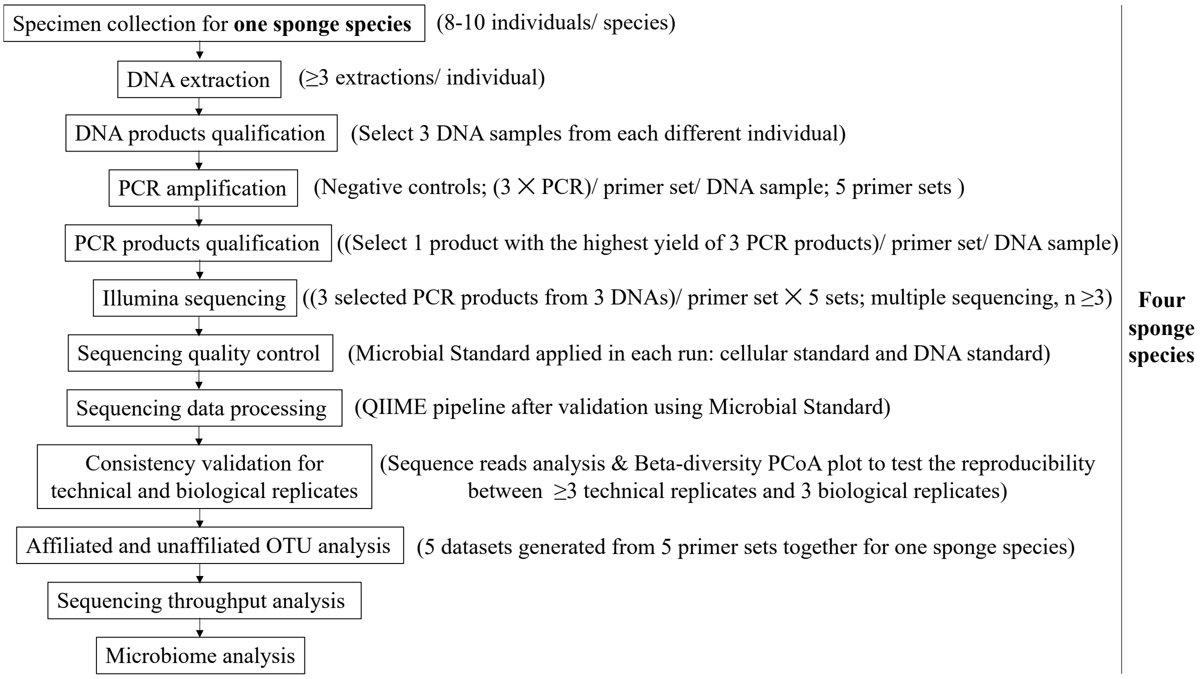


**Supplementary Fig. S5 Experimental design flowchart**

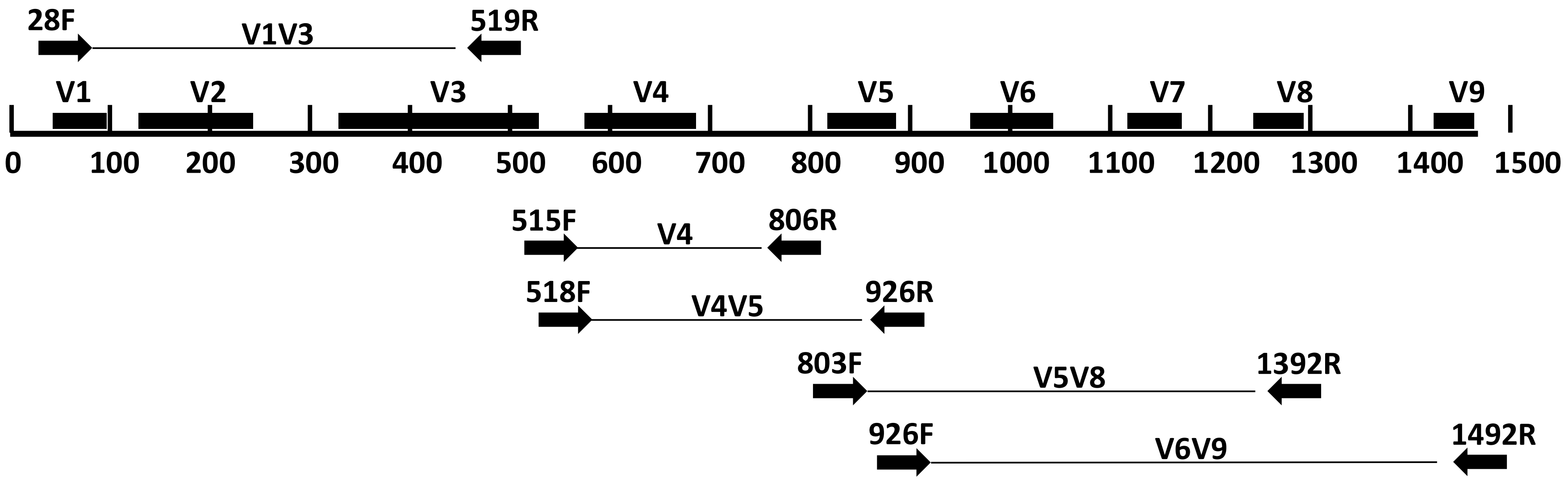


**Supplementary Fig. S6 The position of five region-specific primer sets located at 16S rRNA gene.** The ruler shows the full length of 16S rRNA gene; the black squares represent nine hyper-variable regions V1-V9; the paired arrows mark the specific primer sets with their positions for five selected variable regions in this study.

**Supplementary Table S1. The sequence reads generated by five primer sets for 16S rRNA gene regions V1V3, V4, V4V5, V5V8, and V6V9**

| | Biological Replicate (BR) 1 | | | Biological Replicate 2 | | | Biological Replicate 3 | | | Divergence % | |
|---|---|---|---|---|---|---|---|---|---|---|---|
| | Technical Replicate (TR) 1 | TR 2 | TR 3 | TR 1 | TR 2 | TR 3 | TR 1 | TR 2 | TR 3 | Within BR | Between BR |
| **S2833_V1V3** | 96008 | 96001 | 96009 | 96330 | **96338** | 96329 | 95911 | 95915 | 95918 | **0.007**-0.009 | 0.444 |
| **S2834_V1V3** | 69550 | **69573** | 69539 | 69003 | 69012 | 69009 | 69077 | 69104 | 69099 | 0.013-0.049 | 0.823 |
| **S2835_V1V3** | 80900 | 80901 | 80893 | 80850 | 80854 | 80847 | 80916 | 80910 | **80920** | 0.009-0.012 | 0.090 |
| **S2836_V1V3** | **49392** | 49363 | 49377 | 49118 | 49100 | 49103 | 48961 | 49014 | 49083 | 0.037-**0.249** | **0.877** |
| **S2833_V4** | 100514 | 100529 | **100584** | 100499 | 100510 | 100490 | 100474 | 100477 | 100467 | 0.010-0.070 | 0.116 |
| **S2834_V4** | 100098 | 100075 | 100042 | **100280** | 100201 | 100220 | 99988 | 99966 | 99971 | 0.022-0.079 | 0.317 |
| **S2835_V4** | 100005 | 100010 | 100027 | 100117 | 100131 | **100166** | 100094 | 100072 | 100043 | 0.022-0.050 | 0.161 |
| **S2836_V4** | 41902 | 41914 | 41933 | 41997 | 41958 | 41940 | **42069** | 42038 | 42002 | 0.074-0.160 | 0.398 |
| **S2833_V4V5** | 171900 | 171874 | 171866 | 171883 | 171860 | 171849 | 171993 | **172013** | 171997 | 0.012-0.020 | 0.095 |
| **S2834_V4V5** | 55424 | **55452** | 55419 | 55380 | 55366 | 55349 | 55305 | 55311 | 55324 | 0.034-0.060 | 0.265 |
| **S2835_V4V5** | 96689 | 96666 | 96640 | 96729 | **96798** | 96761 | 96611 | 96636 | 96642 | 0.032-0.071 | 0.193 |
| **S2836_V4V5** | 44529 | 44541 | **44587** | 44499 | 44477 | 44480 | 44500 | 44528 | 44534 | 0.049-0.130 | 0.247 |
| **S2833_V5V8** | 298781 | 298814 | 298825 | 298879 | 298833 | **298920** | 298777 | 298800 | 298810 | 0.011-0.029 | **0.048** |
| **S2834_V5V8** | **641381** | 641299 | 641301 | 641211 | 641247 | 641288 | 641000 | 641037 | 641055 | 0.009-0.013 | 0.059 |
| **S2835_V5V8** | 162099 | 162123 | 162155 | 162172 | 162190 | 162144 | **162256** | 162248 | 162200 | 0.028-0.035 | 0.097 |
| **S2836_V5V8** | 129740 | 129727 | 129750 | 129777 | 129731 | 129750 | 129800 | 129787 | **129829** | 0.018-0.035 | 0.079 |
| **S2833_V6V9** | 57100 | 57122 | 57139 | **57184** | 57153 | 57129 | 57097 | 57105 | 57111 | 0.025-0.096 | 0.152 |
| **S2834_V6V9** | 47800 | 47818 | 47840 | 47833 | 47849 | 47860 | 47828 | **47892** | 47844 | 0.056-0.134 | 0.192 |
| **S2835_V6V9** | 66000 | 66011 | 66034 | **66088** | 66044 | 66022 | 66050 | 66039 | 66068 | 0.044-0.100 | 0.133 |
| **S2836_V6V9** | 45988 | **46002** | 45961 | 45950 | 45936 | 45957 | 45955 | 45968 | 45970 | 0.033-0.089 | 0.144 |

**Supplementary Table S2.** Sequence abundance (%) of Bacterial and Archaeal OTUs at the phylum and class levels revealed by five primer sets.

| | V1V3 | | V4 | | V4V5 | | V5V8 | | V6V9 [d] | |
|---|---|---|---|---|---|---|---|---|---|---|
| | **Bacteria [a]** | **Archaea [b]** | **Bacteria** | **Archaea** | **Bacteria** | **Archaea** | **Bacteria** | **Archaea** | **Bacteria** | **Archaea** |
| Affiliated phyla | 84.61 | 0.01 | 88.47 | 0 | 84.03 | 1.99 | 84.25 | 1.35 | 98.74 | 0.08 |
| Candidate phyla | 1.91 | 0 | 2.78 | 0 | 5.01 | 0 | 2.66 | 0 | 1.18 | 0 |
| Unassigned phyla | 0.69 | 0 | 0.01 | 0 | 0.02 | 0 | 0.17 | 0 | 0 | 0 |
| Unknown phyla | 12.78 | | 8.74 | | 8.95 | | 11.57 | | 0 | |
| Affiliated classes | 71.29[c] | 0.01[c] | 78.98 | 0 | 69.55 | 1.99 | 78.04 | 1.35 | 98.10[e] | 0.08[e] |
| Candidate classes | 13.87 | 0 | 9.48 | 0 | 14.74 | 0 | 5.81 | 0 | 1.64 | 0 |
| Unassigned classes | 2.05 | 0 | 2.80 | 0 | 4.77 | 0 | 3.23 | 0 | 0.18 | 0 |
| Unknown classes | 12.78 | | 8.74 | | 8.95 | | 11.57 | | 0 | |

[a] Distribution of bacterial OTUs at phylum-level, including Affiliated OTUs, Candidate OTUs, Unassigned OTUs, and one Unknown OTUs; [b] Distribution of archaeal OTUs at phylum level is same as bacterial OTUs at phylum level; [c] Distribution of bacterial and archaeal OTUs at class level is same as the one at phylum level; [d] Eukaryote OTUs (0.84%) at phylum level revealed by V6V9 primer set was excluded, the distribution was recalculated based on the sequence reads; [e] Eukaryote OTUs (0.84%) at class level revealed by V6V9 primer set was excluded, the distribution was recalculated based on the sequences reads.

**Supplementary Table S3.** Sequence abundance (%) of phylum and class level OTUs revealed by five primer sets.

**Phylum level OTUs**

| | *Aplysina archeri* | | | *Halichondria okadai* | | | *Igernella notabilis* | | | *Tedania tubulifera* | | | Combined | | |
|---|---|---|---|---|---|---|---|---|---|---|---|---|---|---|---|
| **Primer set** | **Affiliated** | **Candidate** | **Unassigned[a]** | **Affiliated** | **Candidate** | **Unassigned** | **Affiliated** | **Candidate** | **Unassigned** | **Affiliated** | **Candidate** | **Unassigned** | **Affiliated** | **Candidate** | **Unassigned** |
| **V1V3** | **71.82** | **5.10** | **23.08** | **87.16** | **0.07** | **12.77** | **95.14** | **0.26** | **4.60** | **88.75** | **1.02** | **10.23** | **84.62** | **1.92** | **13.47** |
| **V4** | **77.16** | **8.83** | **14.01** | **89.87** | **0.11** | **10.02** | **97.07** | **0.27** | **2.66** | **91.67** | **0.68** | **7.65** | **88.47** | **2.78** | **8.75** |
| **V4V5** | **76.17** | **10.31** | **13.52** | **87.79** | **0.08** | **12.13** | **97.79** | **0.36** | **1.85** | **96.44** | **0.75** | **2.81** | **86.04** | **5.01** | **8.95** |
| **V5V8** | **67.56** | **10.17** | **22.27** | **90.04** | **0.10** | **9.86** | **92.78** | **0.31** | **6.91** | **96.27** | **0.94** | **2.79** | **85.60** | **2.65** | **11.74** |
| **V6V9** | **99.77** | **0.23** | **0.00** | **99.70** | **0.30** | **0.00** | **99.51** | **0.49** | **0.00** | **99.86** | **0.14** | **0.00** | **99.69** | **0.31** | **0.00** |
| **Combined** | **74.02** | **8.57** | **17.41** | **90.16** | **0.11** | **9.73** | **95.83** | **0.33** | **3.84** | **95.01** | **0.77** | **4.22** | **87.62** | **2.30** | **10.08** |

**Class level OTUs**

| | *Aplysina archeri* | | | *Halichondria okadai* | | | *Igernella notabilis* | | | *Tedania tubulifera* | | | Combined | | |
|---|---|---|---|---|---|---|---|---|---|---|---|---|---|---|---|
| **Primer set** | **Affiliated** | **Candidate** | **Unassigned[b]** | **Affiliated** | **Candidate** | **Unassigned** | **Affiliated** | **Candidate** | **Unassigned** | **Affiliated** | **Candidate** | **Unassigned** | **Affiliated** | **Candidate** | **Unassigned** |
| **V1V3** | **32.38** | **40.82** | **26.80** | **86.86** | **0.28** | **12.86** | **94.05** | **1.14** | **4.81** | **87.95** | **1.35** | **10.70** | **71.29** | **13.88** | **14.83** |
| **V4** | **46.45** | **30.43** | **23.12** | **89.45** | **0.43** | **10.12** | **96.23** | **0.89** | **2.88** | **90.65** | **1.45** | **7.90** | **78.97** | **9.48** | **11.55** |
| **V4V5** | **46.08** | **30.45** | **23.47** | **87.40** | **0.41** | **12.19** | **96.88** | **1.01** | **2.11** | **95.24** | **1.65** | **3.11** | **71.57** | **14.73** | **13.71** |
| **V5V8** | **44.86** | **22.17** | **32.97** | **89.40** | **0.30** | **10.30** | **90.89** | **0.78** | **8.33** | **95.17** | **1.75** | **3.08** | **79.40** | **5.82** | **14.78** |
| **V6V9** | **99.17** | **0.73** | **0.10** | **98.79** | **1.04** | **0.17** | **98.71** | **0.93** | **0.36** | **99.80** | **0.18** | **0.02** | **99.08** | **0.74** | **0.18** |
| **Combined** | **47.95** | **26.09** | **25.96** | **89.58** | **0.36** | **10.06** | **94.61** | **0.92** | **4.47** | **94.11** | **1.40** | **4.49** | **80.67** | **6.87** | **12.46** |

[a] The ‘Unaffiliated’ phylum-level OTUs comprise two types of OTUs - Candidate’ and ‘Unassigned’ OTUs; [b] The ‘Unaffiliated’ class-level OTUs comprise two types of OTUs - Candidate’ and ‘Unassigned’ OTUs.

**Supplementary Table S4.** Comparison of the affiliated and unaffiliated phylum-level OTUs between the microbial profiles of four sponge species and 81 species. The number of affiliated and unaffiliated phylum-level OTUs of 81 sponge species refer to the study of [1].

| Affiliated OTUs (phylum) | 4 species V4 | 4 species Combined primer sets | 81 species V4 | Unaffiliated OTUs (phylum) | 4 species V4 | 4 species Combined primer sets | 81 species V4 |
|---|---|---|---|---|---|---|---|
| Crenarchaeota | - | + | + | Bacteria; AncK6 | + | + | + |
| Euryarchaeota | - | + | + | Bacteria; BHI80-139 | + | + | - |
| Acidobacteria | + | + | + | Bacteria; BRC1 | + | + | - |
| Actinobacteria | + | + | + | Bacteria; FBP | + | + | - |
| Aquificae | - | + | - | Bacteria; GN02 | + | + | + |
| Armatimonadetes | + | + | + | Bacteria; GN04 | + | + | + |
| Bacteroidetes | + | + | + | Bacteria; GOUTA4 | - | + | - |
| Caldithrix | - | + | + | Bacteria; KSB3 | + | + | - |
| Chlamydiae | - | + | + | Bacteria; LD1 | + | + | - |
| Chlorobi | - | + | + | Bacteria; MVS-104 | - | + | - |
| Chloroflexi | + | + | + | Bacteria; NC10 | + | + | - |
| Cyanobacteria | + | + | + | Bacteria; NKB19 | + | + | - |
| Elusimicrobia | - | + | + | Bacteria; OD1 | + | + | + |
| Firmicutes | + | + | + | Bacteria; OP1 | - | - | + |
| Fusobacteria | + | + | + | Bacteria; OP11 | + | + | - |
| Gemmatimonadetes | + | + | + | Bacteria; OP3 | + | + | - |
| Lentisphaerae | + | + | + | Bacteria; OP8 | - | + | + |
| Nitrospirae | + | + | + | Bacteria; Other | + | + | - |
| Parvarchaeota | - | - | + | Bacteria; PAUC34f | + | + | + |
| Planctomycetes | + | + | + | Bacteria; SAR406 | + | + | + |
| Poribacteria | + | + | + | Bacteria; SBR1093 | + | + | + |
| Proteobacteria | + | + | + | Bacteria; SR1 | + | + | - |
| Spirochaetes | + | + | + | Bacteria; TM6 | + | + | + |
| Synergistetes | + | + | - | Bacteria; TM7 | + | + | - |
| Tenericutes | - | + | + | Bacteria; Unassigned | + | + | - |
| Thermi | + | + | + | Bacteria; WPS-2 | + | + | + |
| Thermotogae | - | + | - | Bacteria; WS2 | + | + | + |
| Verrucomicrobia | + | + | + | Bacteria; WS3 | + | + | + |
| | | | | Bacteria; WS5 | - | - | + |
| | | | | Bacteria; WWE1 | - | + | - |
| | | | | Bacteria; ZB3 | + | + | + |
| | | | | Unassigned;Other | + | + | + |

| Affiliated OTUs (class in phylum Proteobacteria) | 4 species V4 | 4 species Combined primer sets | 81 species V4 | Unaffiliated OTUs (class in phylum Proteobacteria) | 4 species V4 | 4 species Combined primer sets | 81 species V4 |
|---|---|---|---|---|---|---|---|
| Alphaproteobacteria | + | + | + | Proteobacteria; Other | + | + | - |
| Betaproteobacteria | + | + | + | Proteobacteria; TA18 | + | + | + |
| Deltaproteobacteria | + | + | + | Proteobacteria; unassigned | + | + | + |
| Epsilonproteobacteria | + | + | + | | | | |
| Gammaproteobacteria | + | + | + | | | | |
| Zetaproteobacteria | - | + | - | | | | |

**Supplementary Table S5. The microbiota coverage of five region-specific primer sets by in silico PCR evaluation using SILVA database [a]**

| Region-specific primer set | Domain | Phylum | Order | Class | Family | Genus |
|---|---|---|---|---|---|---|
| V1V3 | 4 | 81 | 163 | 411 | 721 | 3196 |
| V4 | 4 | 91 | 195 | 467 | 813 | 3688 |
| V4V5 | 4 | 247 | 508 | 1094 | 1239 | 7722 |
| V5V8 | 4 | 166 | 577 | 1317 | 1626 | 9706 |
| V6V9 | 4 | 22 | 32 | 40 | 37 | 36 |

[a]The evaluation was conducted in online tool TestPrime 1.0 (https://www.arb-silva.de/search/testprime/) under the settings of RefNR (more uniform distribution of sequences across taxa than Ref) as the Sequence Collection with zero maximum number of mismatches, and SILVA Ref NR as taxonomic annotation reference.

**Supplementary Table S6.** 16S rRNA gene region-specific primer sets for metagenomic sequencing

| Region-specific Primer set | Target region | Forward &Reverse primer Sequences | Reference |
|---|---|---|---|
| 28F/519R | V1V3 | 5'-GAGTTTGATCNTGGCTCAG<br>5'-GTNTTACNGCGGCKGCTG | 42 |
| 515F/806R | V4 | 5'-GTGYCAGCMGCCGCGGTAA<br>5'-GGACTACNVGGGTWTCTAAT | 43 |
| 518F/926R | V4V5 | 5'-CCAGCAGCYGCGGTAAN<br>5'-CCGTCAATTCNTTTRAGT | 44 |
| 803F [a]/1392R | V5V8 | 5'-TTAGANACCCNNGTAGTC<br>5'-ACGGGCGGTGWGTRC | 17 |
| 926F/1492R | V6V9 | 5'-CCGTCAATTCNTTTRAGT<br>5′-GGTTACCTTGTTACGACTT | 44, 45 |

[a] primer 803F is modified by mixing 4 primers
